# Supplementary material for: mTOR inhibition attenuates chemosensitivity through the induction of chemotherapy resistant persisters
Source: Nat Commun. 2022 Nov 17;13:7047. doi: 10.1038/s41467-022-34890-6 (PMC9671908; doi:10.1038/s41467-022-34890-6)
Supplement: Supplementary file 1 — Supplementary Information [file 41467_2022_34890_MOESM1_ESM.pdf]

Supplementary Information

mTOR Inhibition Attenuates Chemosensitivity through the Induction of Chemotherapy Resistant Persisters

Yuanhui Liu, Nancy G. Azizian, Delaney K. Sullivan, Yulin Li

Supplementary Figure 1

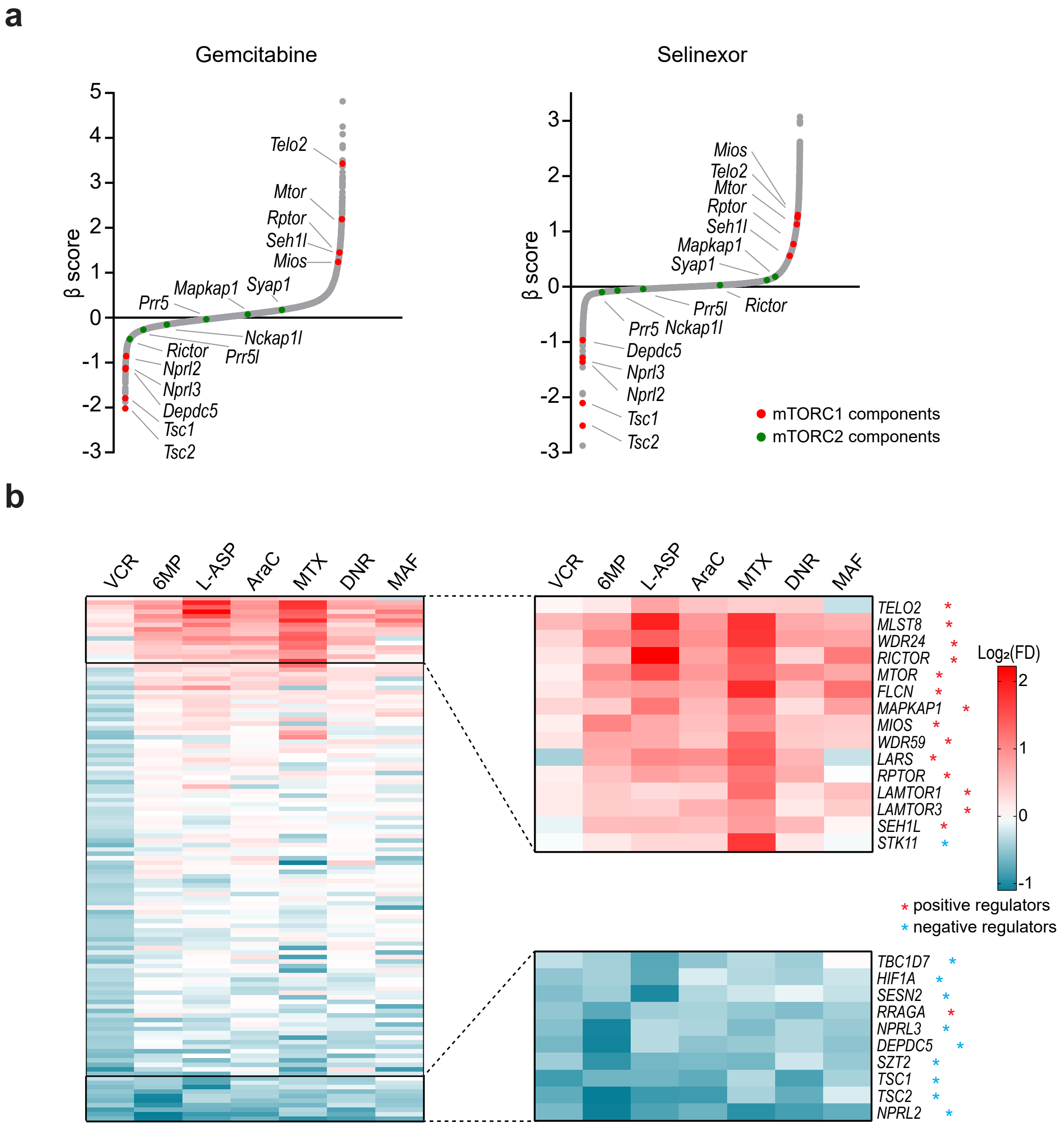

Supplementary Fig. 1. Enrichment of positive, and depletion of negative regulators of mTOR signaling in CRISPR library screening of DNA damaging agents. a Gene ranking of the mTORC1 *vs* mTORC2 components in gemcitabine and selinexor screens based on β scores. **b** Reanalysis of the CRISPR screen reported by Oshima K et al[1], performed in REH acute lymphoblastic leukemia cells carrying a TP53 mutation. Cells were treated with seven chemotherapeutic agents (VCR: vincristine; 6MP: 6-mercaptopurine; L-ASP: L-asparaginase; AraC: cytarabine; MTX: methotrexate; DNR: daunorubicin; and MAF: maphosphamide). Red and blue asterisks denote positive and negative regulators of the mTOR signaling pathway. Genes were ranked based on the average log2 fold changes of the seven drugs. Color bar indicates the scale of log2 fold change. Source data are provided as a Source Data file.

Supplementary Figure 2

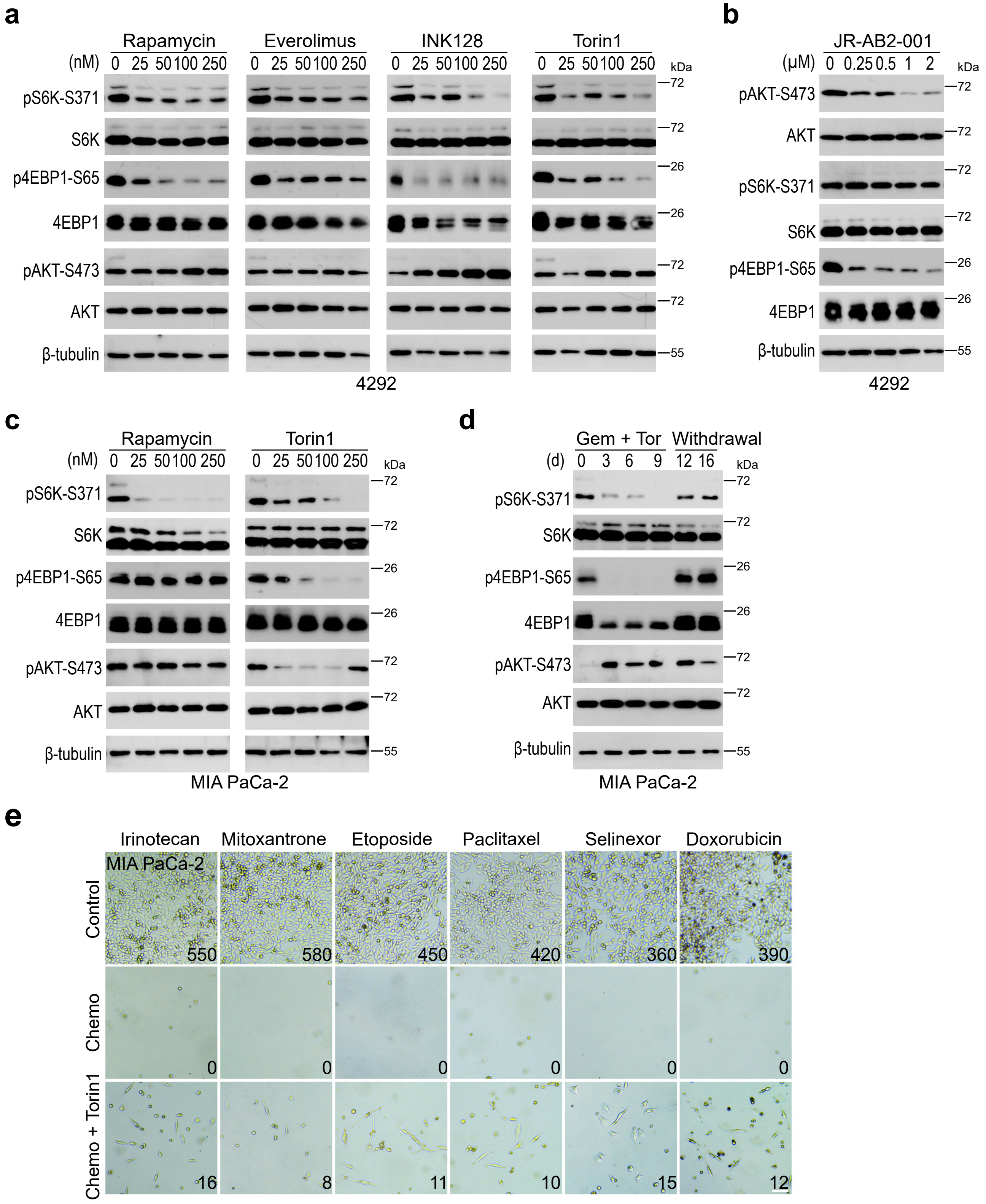

Supplementary Figure 2 *(continued)*

f

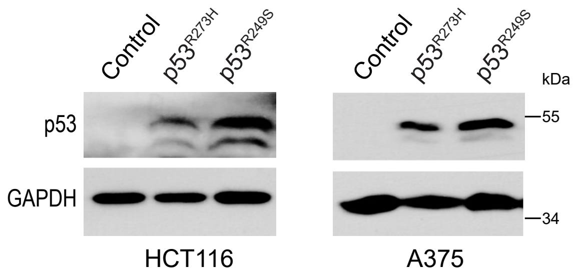

g

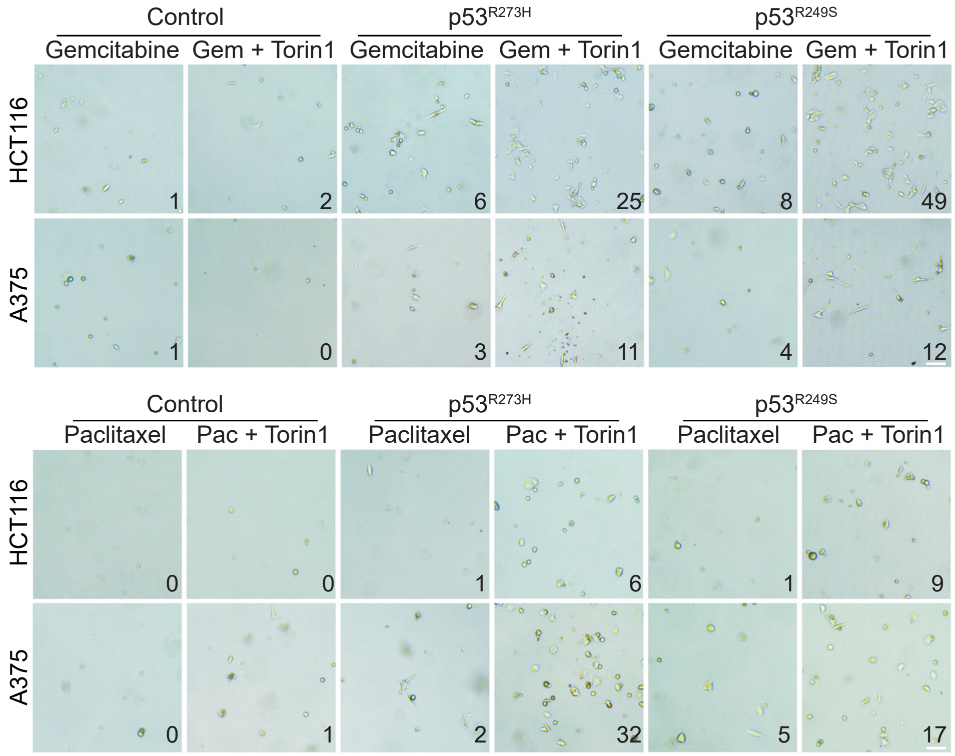

Supplementary Fig. 2. mTOR inhibition induces drug-tolerant persisters. a Efficiency of mTOR inhibitors as shown by Western blot analysis. 4292 cells were treated with 0-250nM rapamycin, everolimus, INK128, and Torin1 for seven days. mTORC1 (phospho-S6K-S371 and phospho-4EBP1-S65) and mTORC2 (phospho-AKT-S473) activities were detected by Western blot. Experiments in Supplementary Fig. 2a-d, g were repeated once with similar results. **b** Activity of JR-AB2-001 in 4292 cells. Cells were treated with 0-2μM JR-AB2-001 for seven days and mTORC1 and mTORC2 activities were detected by Western blot. **c** mTORC1 and mTORC2 activities were detected by Western blot in MIA PaCa-2 cells treated with 0-250nM rapamycin or Torin1 for nine days. **d** MIA PaCa-2 cells were treated with 100nM gemcitabine plus 100nM Torin1 for three, six, and nine days followed by drug withdrawal (day 12 and 16). mTORC1 and mTORC2 activities were detected by Western blot. **e** Representative brightfield images of MIA PaCa-2 cells treated with chemotherapeutic agents (100nM doxorubicin, 2μM selinexor, 10nM paclitaxel, 6μM etoposide, 60nM mitoxantrone, and 6μM irinotecan), in the presence or absence of 100nM Torin1 for nine days. Experiments were performed with three biological replicates. The average cell count per image is indicated in the lower right corner. Scale bar, 100μm. **f** Western blot analysis of the ectopic expression of p53 mutants in HCT116 and A375 cells[2]. GAPDH serves as the loading control. **g** Representative images of HCT116 and A375 cells expressing p53 mutants treated with chemotherapy or chemotherapy plus 100nM Torin1 for nine days. HCT116 cells were treated with 3μM gemcitabine and 100nM paclitaxel, and A375 cells were treated with 1μM gemcitabine and 10nM paclitaxel. The average cell count per image is indicated in the lower right corner. Experiments were performed in biological triplicates. Scale bar, 100μm.

Supplementary Figure 3

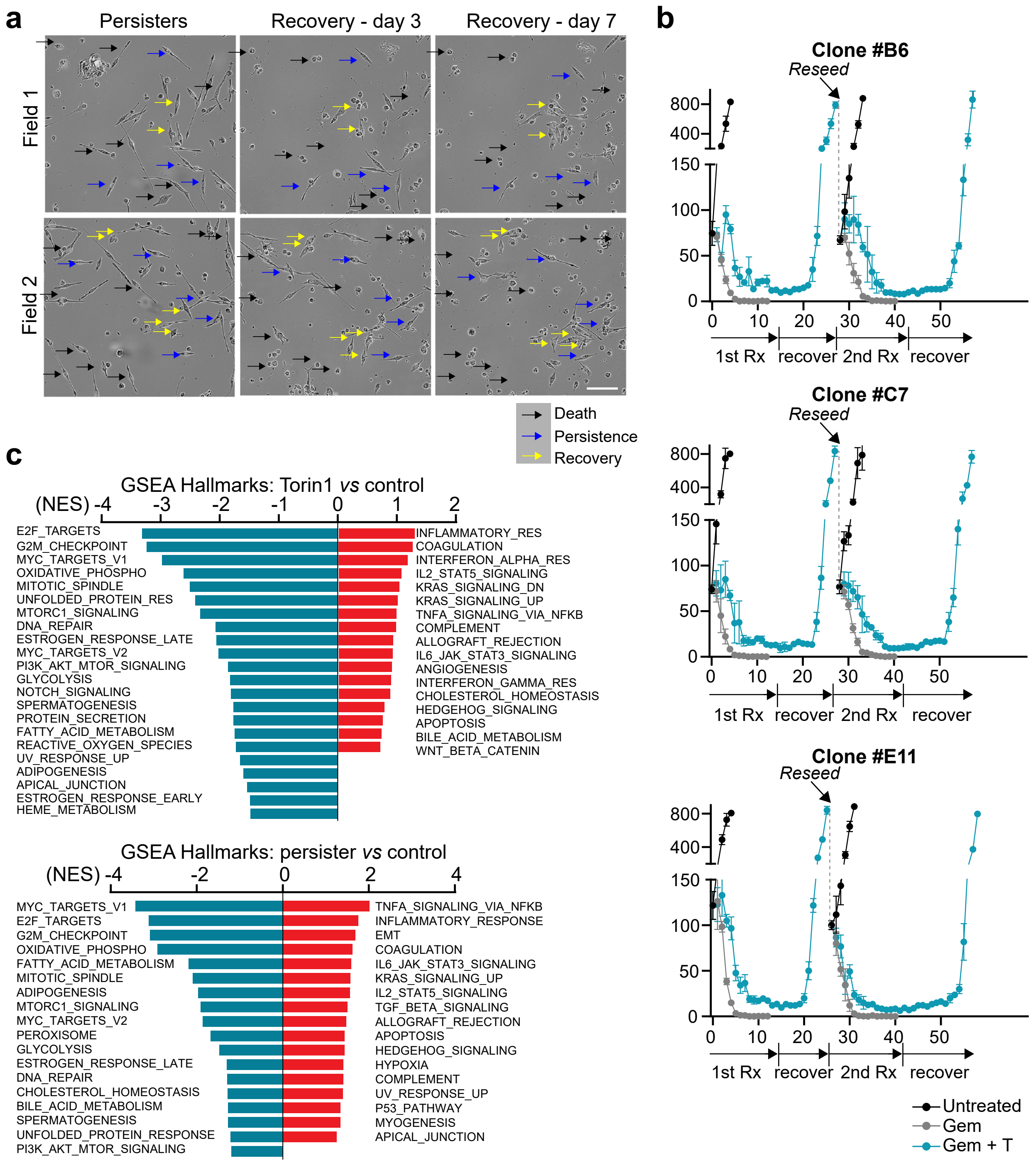

Supplementary Fig. 3. The persister phenotype is reversible. a Representative images of persister recovery following drug removal. Persister cell proliferation and death were tracked in the same bright fields for seven days. Experiment was performed with three biological replicates. Scale bar, 100μm. **b** Reversibility of persister phenotype in three independent single-cell clones derived from MIA PaCa-2. Y axis represents the average number of cells in a 50X field (area of 0.64 mm^2). For each data point, cells from at least three independent fields were counted and cell numbers were plotted as mean ± SEM. **c** GSEA results using the MSigDB hallmark gene sets in persisters *vs* control, and Torin1-treated *vs* control pairs. Normalized enrichment scores (NES). Source data are provided as a Source Data file.

Supplementary Figure 4

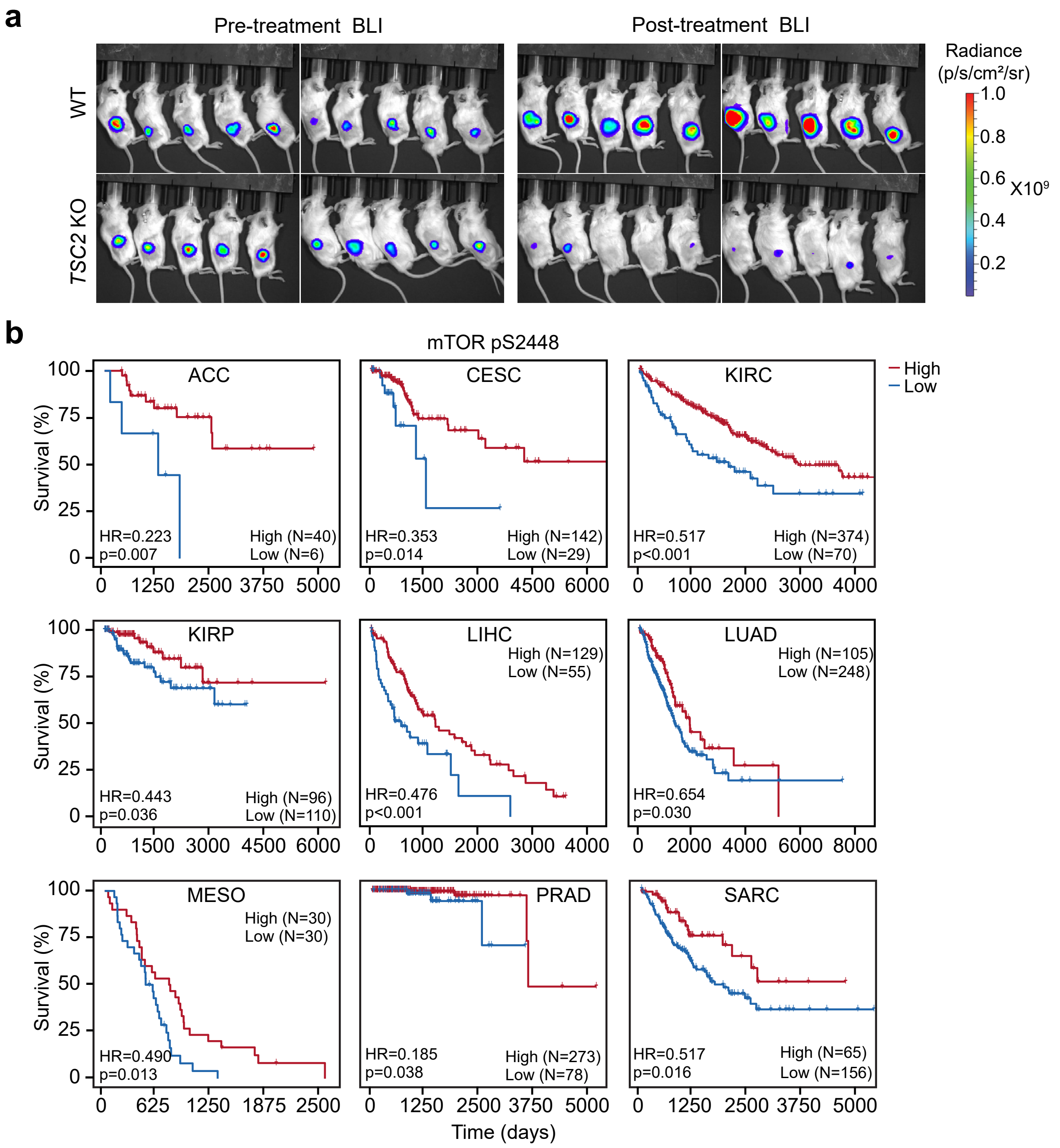

Supplementary Fig. 4. mTOR activation increases chemosensitivity. a Representative BLI images showing the tumor size before and after four weeks of gemcitabine treatment. **b** Survival analysis of various human cancer patients based on phospho-mTOR-S2448 RPPA data. Data were derived from The Cancer Proteome Atlas and analyzed using the TRGAted application[3,4]. Y axis indicates the fraction of survival, while X axis shows days of follow-up. The number of patients (N) in phospho-mTOR-S2448 high and low groups for each cancer type is: Adrenocortical carcinoma (ACC), N = 40, 6; Cervical squamous cell carcinoma and endocervical adenocarcinoma (CESC), N = 142, 29; Kidney renal clear cell carcinoma (KIRC), N = 374, 70; Kidney renal papillary cell carcinoma (KIRP), N = 96, 110; Liver hepatocellular carcinoma (LIHC), N = 129, 55; Lung adenocarcinoma (LUAD), N = 105, 248; Mesothelioma (MESO), N = 30, 30; Prostate adenocarcinoma (PRAD), N = 273, 78; Sarcoma (SARC), N = 65, 156. Log-rank (Mantel-Cox) test.

Supplementary Figure 5

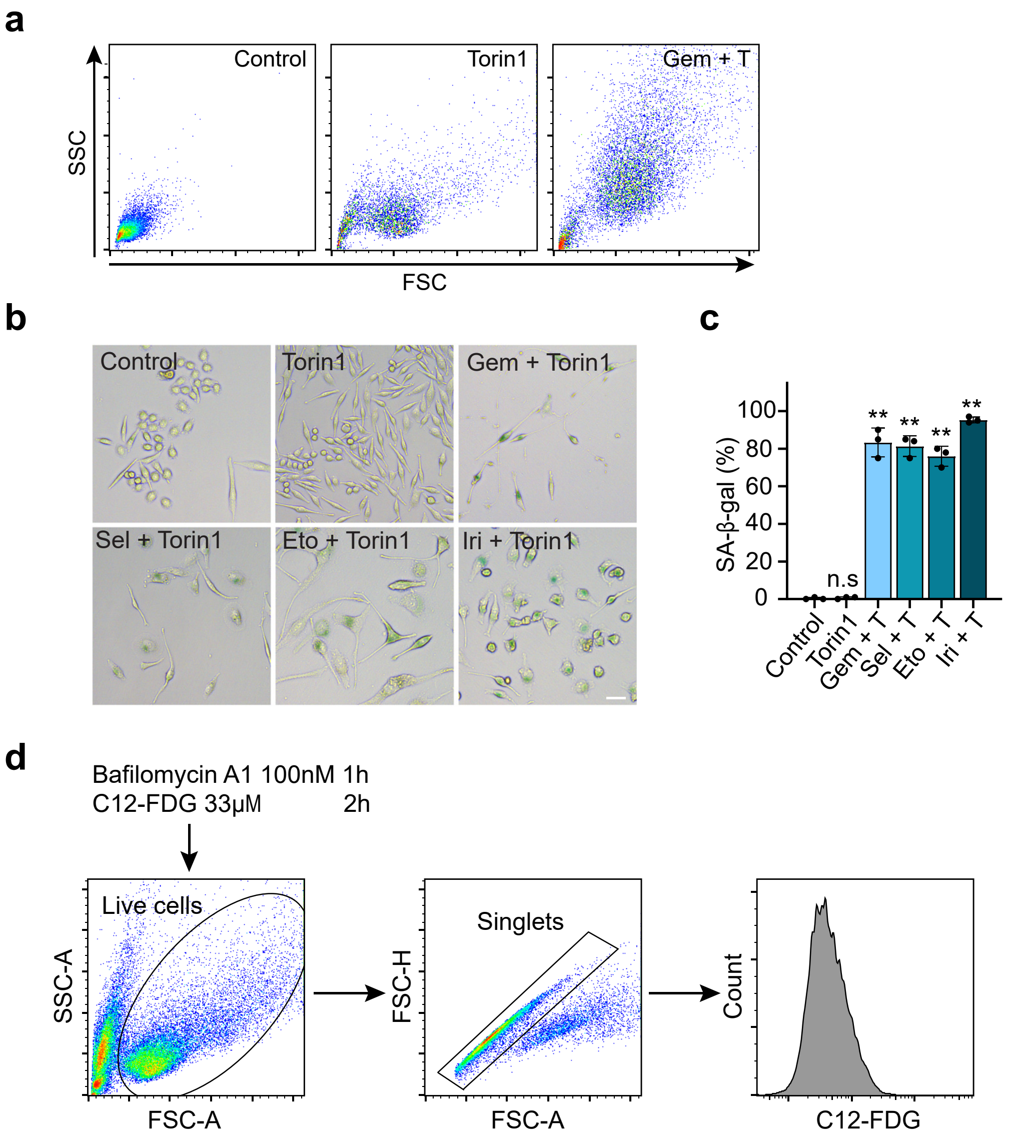

Supplementary Fig. 5. The senescence phenotype of persisters. a Flow cytometric analysis of FSC and SSC in MIA PaCa-2 cells treated with 100nM Torin1 or Torin1 plus 100nM gemcitabine for nine days. Experiments were performed at least three times with similar results. **b-c** SABG staining and quantification of MDA-Panc-28 cells treated with 100nM Torin1, or Torin1 plus chemotherapeutic agents for six days (10μM gemcitabine, 1μM selinexor, 3μM etoposide, and 10μM irinotecan). Data were plotted as mean ± SEM. Experiments were performed with three biological replicates. Two-tailed Student's T test. ** $p < 0.001$, n.s: not significant. The p values for the Gem + T, Sel + T, Eto + T, and Iri + T treatments *vs* control comparisons are 4.74e-07, 1.44e-04, 1.61e-05, 5.81e-08. Scale bar, 50μm. Source data are provided as a Source Data file. **d** Gating strategy for the flow cytometric quantification of SABG activity shown in Fig. 4c-d.

Supplementary Figure 6

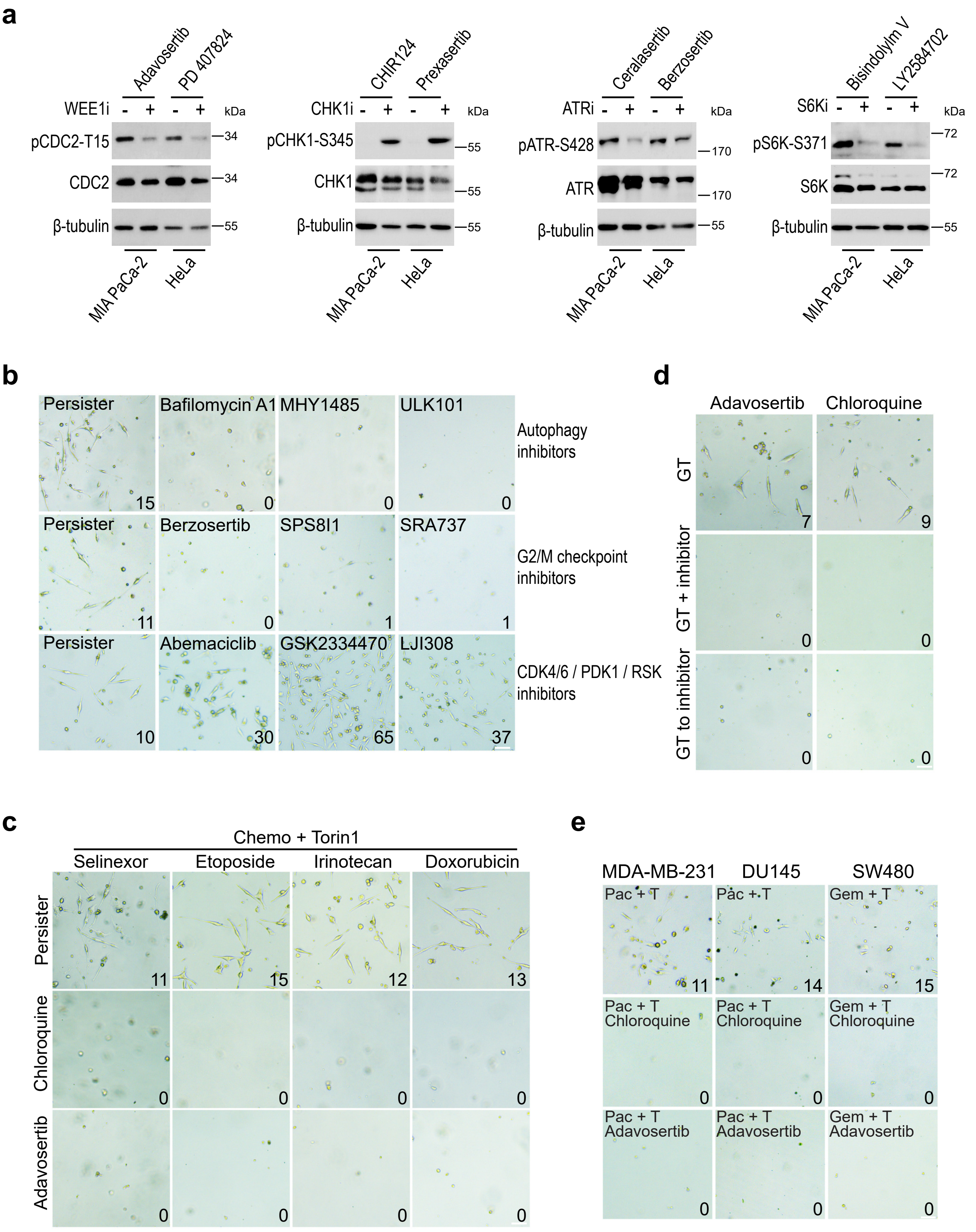

Supplementary Fig. 6. A small-molecule chemical library screen reveals the survival mechanisms of persisters. a Western blot analysis of WEE1, CHK1, ATR, and S6K inhibition. MIA PaCa-2 cells were treated with 100nM adavosertib (WEE1i), 100nM CHIR124 (CHK1i), 10μM ceralasertib (ATRi) and 30μM bisindolylmaleimide V (S6Ki) for nine days. HeLa cells were treated with 3μM PD 407824 (WEE1i), 1μM prexasertib HCl (CHK1i), 300nM berzosertib (ATRi) and 1μM LY2584702 (S6Ki) for seven days. Phospho-CDC2-T15, phospho-CHK1-S345, phospho-ATR-S428, and phospho-S6K-S371 were detected by Western blot. β-tubulin serves as the loading control. Experiment was repeated once with similar results. **b** Representative images of MIA PaCa-2 cells treated with 100nM gemcitabine and 100nM Torin1, plus autophagy inhibitors (2nM bafilomycin A1, 20μM MHY1485, 20μM ULK101), G2/M checkpoint inhibitors (100nM berzosertib, 300nM SPS8I1, 3μM SRA737), and inhibitors for CDK4/6 (600nM abemaciclib), PDK1 (3μM GSK2334470), and RSK (30μM LJI308). Experiments were performed with three biological replicates. The average cell count per image is indicated in the lower right corner. Scale bar, 100μm. **c** Representative images of MIA PaCa-2 cells treated with various chemotherapeutic agents (2μM selinexor, 6μM etoposide, 6μM irinotecan and 100nM doxorubicin) plus 100nM Torin1, or in combination with 10μM chloroquine or 100nM adavosertib for nine days. The experiments were performed with three biological replicates. Scale bar, 100μm. **d** Representative images of MIA PaCa-2 cells treated with 100nM gemcitabine and 100nM Torin1 (GT) (top panel), with 100nM adavosertib or 10μM chloroquine added at the beginning of GT treatment (GT + inhibitor) (middle panel), or five days after the start of GT treatment (GT to inhibitor) (bottom panel). Experiments were performed with three biological replicates. Scale bar, 100μm. **e** Representative images of MDA-MB-231, DU145, and SW480 persisters following treatment with chloroquine or adavosertib. Persisters were induced in MDA-MB-231 by treatment with 10nM paclitaxel and 100nM Torin1 for nine days, DU145 with 10nM paclitaxel and 300nM Torin1 for seven days, and SW480 with 1μM gemcitabine and 300nM Torin1 for nine days. Chloroquine or adavosertib were added at the beginning of persister-induction treatment. Experiments were performed with three biological replicates. Scale bar, 100μm.

Supplementary Figure 7

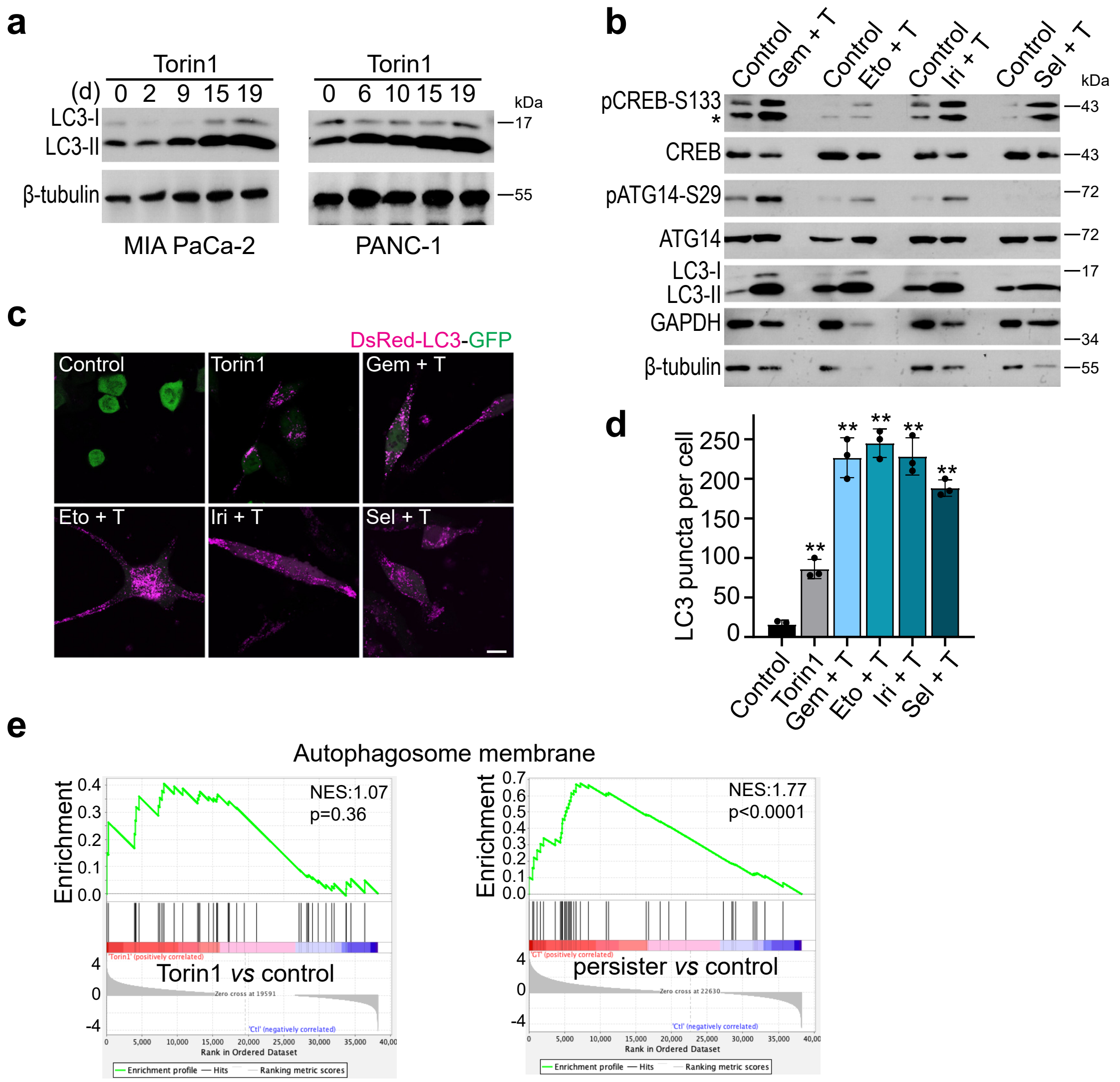

Supplementary Fig. 7. Induction of autophagy in persisters. a LC3 induction in MIA PaCa-2 and PANC-1 cells following treatment with 100nM Torin1 for the indicated time. Experiment was repeated once with similar results. **b** Western analysis of phospho-CREB-S133, phospho-ATG14-S29, and LC3 in persister cells treated with Torin1 plus chemotherapeutic agents (100nM gemcitabine, 2μM selinexor, 6μM etoposide, and 6μM irinotecan) for nine days. Asterisk indicates a non-specific signal. Experiment was repeated once with similar results. **c-d** Representative images and quantification of LC3 puncta in MIA PaCa-2 treated with 100nM Torin1 or Torin1 plus chemotherapeutic agents (100nM gemcitabine, 2μM selinexor, 6μM etoposide, and 6μM irinotecan) for nine days. Data were plotted as mean ± SEM. Experiments were performed in biological triplicates. Two-tailed Student's T test. ** $p < 0.001$. The p values for the Torin1, Gem + T, Sel + T, Eto + T, and Iri + T treatments *vs* control comparisons are 7.95e-04, 1.43e-04, 2.97e-05, 1.09e-04, and 1.39e-05. Scale bar, 5μm. **e** GSEA of autophagy signature (GOCC_AUTOPHAGOSOME_MEMBRANE from MSigDB) in our RNA-seq study of the Torin1-treated and persister cells. Normalized enrichment scores (NES) and nominal p values are shown. Kolmogorov-Smirnov test, $p = 0.36$ (Torin1 *vs* control), $p < 0.0001$ (persister *vs* control). Source data are provided as a Source Data file.

Supplementary Figure 8

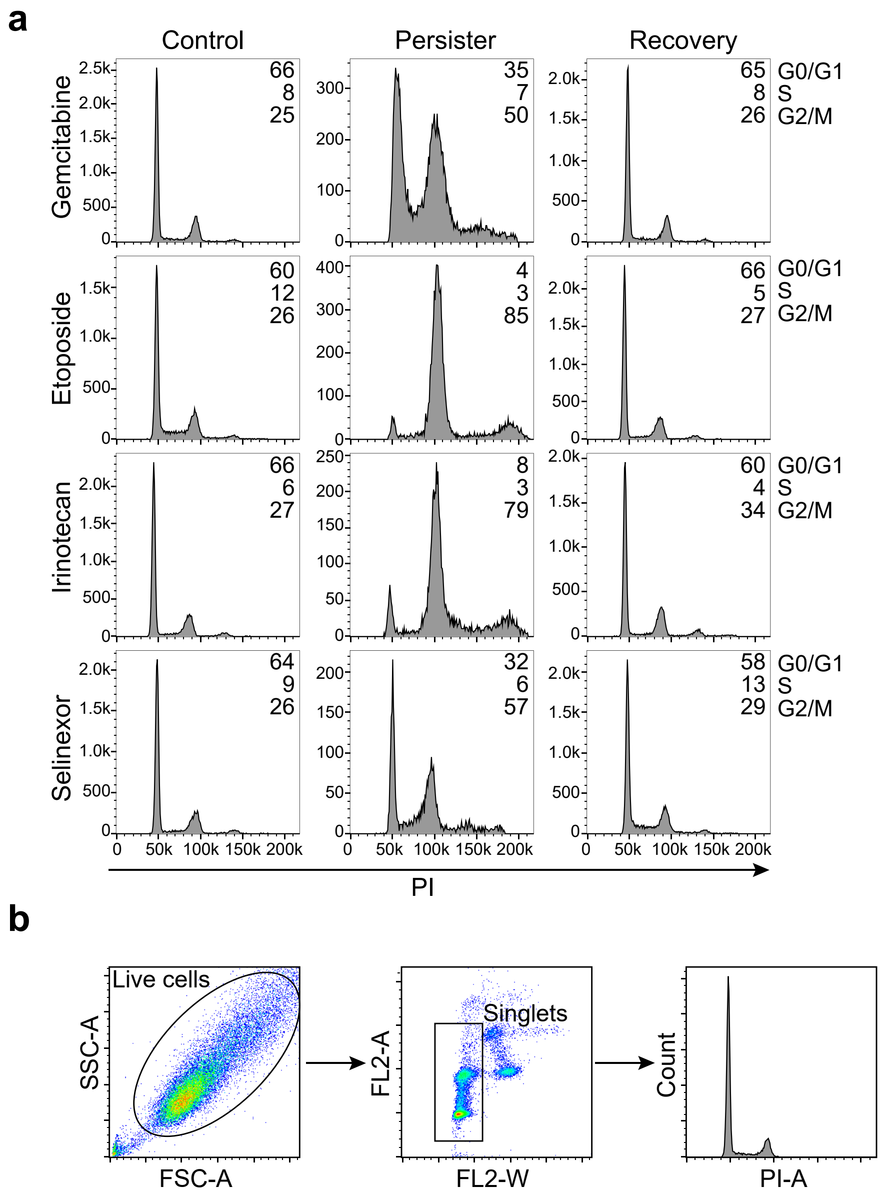

Supplementary Fig. 8. G2/M checkpoint activation and cell cycle arrest in persisters. a Flow cytometric analysis of the cell cycle following propidium iodide (PI) staining in control, persister, and recovered cells. The percentage of cells in different phases of the cell cycle was labeled in the top right corner. MIA PaCa-2 cells were treated with Torin1 plus chemotherapeutic agents (100nM gemcitabine, 2μM selinexor, 6μM etoposide, and 6μM irinotecan) for nine days (persisters) and cultured in drug free media for an additional 9-12 days (recovered cells). **b** Gating strategy for the cell cycle analysis shown in Supplementary Fig. 8a.

Supplementary Table 1. Summary of persister phenotypes in a panel of 29 human cancer cell lines from diverse tissue origins. Cells were treated with chemotherapy or chemotherapy plus Torin1. Experimental details are listed in Supplementary Data 2. Experiments were performed in biological duplicates.

**Pancreatic cancer**	**TP53 status**	**Gemcitabine**	**Irinotecan**	**Etoposide**
MDA-Panc-28	Mutant	+	+	+
BxPC-3	Mutant	+	+	+
AsPC-1	Mutant	-	+	+
Capan-2	WT	+	-	-
**Prostate cancer**	**TP53 status**	**Paclitaxel**	**Mitoxantrone**	**Estramustine**
LNCaP	Mutant	+	-	-
C4-2B	Mutant	+	+	+
DU 145	Mutant	+	+	+
22Rv1	Mutant	+	-	+
PC3	Mutant	+	-	n.d.
**Breast cancer**	**TP53 status**	**Paclitaxel**	**Carboplatin**	**Gemcitabine**
T-47D	Mutant	+	+	+
MCF7	WT	-	-	-
MDA-MB-231	Mutant	+	+	+
SK-BR-3	Mutant	+	+	+
**Liver cancer**	**TP53 status**	**Gemcitabine**	**Doxorubicin**	**Mitoxantrone**
PLC/PRF/5	Mutant	+	+	+
SNU398	Mutant	+	+	+
Hep3B	Mutant	-	-	-
HepG2	WT	-	-	-
**Melanoma**	**TP53 status**	**Paclitaxel**	**Carboplatin**	**Gemcitabine**
Mewo	Mutant	+	+	+
A375	WT	-	n.d.	-
**Colon cancer**	**TP53 status**	**Irinotecan**	**Gemcitabine**	**Etoposide**
HCT116	WT	n.d.	-	-
SW480	Mutant	+	+	+
**Lung cancer**	**TP53 status**	**Etoposide**	**Gemcitabine**	**Irinotecan**
H460	WT	-	-	-
A549	WT	-	-	-
H1299	Mutant	+	+	+
H441	Mutant	+	+	+
**Head and neck cancer**	**TP53 status**	**Paclitaxel**	**Carboplatin**	**Gemcitabine**
SCC47	WT	-	n.d.	-
**Ovarian cancer**	**TP53 status**	**Etoposide**	**Gemcitabine**	**Irinotecan**
SK-OV-3	Mutant	+	+	+
**Cervical cancer**	**TP53 status**	**Etoposide**	**Gemcitabine**	**Irinotecan**
HeLa	Mutant	+	n.d.	+
**Osteosarcoma**	**TP53 status**	**Etoposide**	**Gemcitabine**	**Doxorubicin**
U2OS	WT	+	+	+

Notes:
"+": Persisters induced by treatment with the chemotherapeutic agents plus 100nM Torin1.
"-" : No persisters observed following the combined treatment.
"n.d.": Not determined due to intrinsic resistance to the specific chemotherapeutic agents.

Supplementary References

1 Oshima, K. *et al.* Mutational and functional genetics mapping of chemotherapy resistance mechanisms in relapsed acute lymphoblastic leukemia. *Nat Cancer* **1**, 1113-1127, doi:10.1038/s43018-020-00124-1 (2020).

2 Junk, D. J. *et al.* Different mutant/wild-type p53 combinations cause a spectrum of increased invasive potential in nonmalignant immortalized human mammary epithelial cells. *Neoplasia* **10**, 450-461, doi:10.1593/neo.08120 (2008).

3 Li, J. *et al.* TCPA: a resource for cancer functional proteomics data. *Nat Methods* **10**, 1046-1047, doi:10.1038/nmeth.2650 (2013).

4 Borcherding, N., Bormann, N. L., Voigt, A. P. & Zhang, W. TRGAted: A web tool for survival analysis using protein data in the Cancer Genome Atlas. *F1000Res* **7**, 1235, doi:10.12688/f1000research.15789.2 (2018).
