# Supplementary material for: mTOR inhibition attenuates chemosensitivity through the induction of chemotherapy resistant persisters
Source: Nat Commun. 2022 Nov 17;13:7047. doi: 10.1038/s41467-022-34890-6 (PMC9671908; doi:10.1038/s41467-022-34890-6)
Supplement: Supplementary file 2 — Description of Additional Supplementary Files [file 41467_2022_34890_MOESM2_ESM.pdf]

Description of Additional Supplementary Files

File Name: Supplementary Data 1
Description: Gene enrichment and depletion analysis of gemcitabine and selinexor screens.
Data 1a Gene ranking according to β scores in gemcitabine and selinexor screens.
Data 1b List of genes enriched in both screens.
Data 1c List of genes depleted in both screens.
Data 1d GO term enrichment analysis of enriched genes.
Data 1e GO term enrichment analysis of depleted genes.

File Name: Supplementary Data 2
Description: Experimental conditions for persister testing in a panel of human cancer cell lines.

File Name: Supplementary Data 3
Description: RNA-seq analysis of control, Torin1-treated, persisters, and recovered cells.
Data 3a RNA-seq profiles of control, Torin1-treated, persisters, and recovered cells.
Data 3b Differential gene expression between control and persisters.
Data 3c Differential gene expression between control and recovered cells.
Data 3d Differential gene expression between control and Torin1-treated cells.

File Name: Supplementary Data 4
Description: mTOR-regulated persister signature and GSEA in human patients' residual tumors.
Data 4a Genes included in the mTOR-regulated persister signature.
Data 4b NES, norminal p, and FDR q values for GSEA of public data sets.

File Name: Supplementary Data 5
Description: Pre- and post-treatment BLI data for MIA PaCa-2 WT and TSC2 KO tumors.

File Name: Supplementary Data 6
Description: Chemical library screening to identify mechanisms of persister survival.
Data 6a List of small-molecule chemicals included in the screen.
Data 6b Chemical inhibitors significantly decreasing or increasing persisters.
