# Supplementary material for: mTOR inhibition attenuates chemosensitivity through the induction of chemotherapy resistant persisters
Source: Nat Commun. 2022 Nov 17;13:7047. doi: 10.1038/s41467-022-34890-6 (PMC9671908; doi:10.1038/s41467-022-34890-6)
Supplement: Supplementary file 3 — Reporting Summary [file 41467_2022_34890_MOESM3_ESM.pdf]

nature portfolio

Corresponding author(s): Yulin Li

Last updated by author(s): Nov 2, 2022

Reporting Summary

Nature Portfolio wishes to improve the reproducibility of the work that we publish. This form provides structure for consistency and transparency in reporting. For further information on Nature Portfolio policies, see our Editorial Policies and the Editorial Policy Checklist.

Statistics

For all statistical analyses, confirm that the following items are present in the figure legend, table legend, main text, or Methods section.

n/a	Confirmed	
☐	☒	The exact sample size (*n*) for each experimental group/condition, given as a discrete number and unit of measurement
☐	☒	A statement on whether measurements were taken from distinct samples or whether the same sample was measured repeatedly
☐	☒	The statistical test(s) used AND whether they are one- or two-sided<br>*Only common tests should be described solely by name; describe more complex techniques in the Methods section.*
☐	☒	A description of all covariates tested
☒	☐	A description of any assumptions or corrections, such as tests of normality and adjustment for multiple comparisons
☐	☒	A full description of the statistical parameters including central tendency (e.g. means) or other basic estimates (e.g. regression coefficient) AND variation (e.g. standard deviation) or associated estimates of uncertainty (e.g. confidence intervals)
☐	☒	For null hypothesis testing, the test statistic (e.g. *F*, *t*, *r*) with confidence intervals, effect sizes, degrees of freedom and *P* value noted<br>*Give P values as exact values whenever suitable.*
☒	☐	For Bayesian analysis, information on the choice of priors and Markov chain Monte Carlo settings
☒	☐	For hierarchical and complex designs, identification of the appropriate level for tests and full reporting of outcomes
☒	☐	Estimates of effect sizes (e.g. Cohen's *d*, Pearson's *r*), indicating how they were calculated

Our web collection on statistics for biologists contains articles on many of the points above.

Software and code

Policy information about availability of computer code

Data collection	Cell cycle FUCCI assay and autophagic reporter assay images were acquired using Olympus FV31S software.<br>Multicolor competition assay images were acquired using LAS X (v3.7.4).<br>Flow cytometry samples were acquired using FACS LSRII flow cytometer (BD Bioscience).<br>In vivo BLI was performed on an IVIS Lumina III platform.
Data analysis	Microsoft Excel 2015 and GraphPad Prism v8.4.2 were used for statistics.<br>FUCCI images were analyzed using Olympus CellSens Dimension software.<br>Analysis of the sgRNA libraries was performed using MAGeCK (v0.5.9.2).<br>For RNAseq, the R library, sleuth (version 0.30.0), was used to produce normalized gene-level abundance estimates and perform differential gene expression analysis.<br>Gene Ontology (GO) term enrichment analysis was performed using EnrichR (website tool).<br>Flow cytometry data were analyzed using FlowJo software (v10.8.1).<br>GSEA analysis was performed using the software GSEA_4.1.0 downloaded from http://www.gsea-msigdb.org/gsea/downloads.jsp.<br>In vivo BLI data were analyzed using the Living Image software (v4.2).

For manuscripts utilizing custom algorithms or software that are central to the research but not yet described in published literature, software must be made available to editors and reviewers. We strongly encourage code deposition in a community repository (e.g. GitHub). See the Nature Portfolio guidelines for submitting code & software for further information.

Data

Policy information about availability of data

All manuscripts must include a data availability statement. This statement should provide the following information, where applicable:

- Accession codes, unique identifiers, or web links for publicly available datasets
- A description of any restrictions on data availability
- For clinical datasets or third party data, please ensure that the statement adheres to our policy

Sequencing data generated in this study have been deposited in GEO with accession number GSE162065 [https://www.ncbi.nlm.nih.gov/geo/query/acc.cgi?acc=GSE162065] for the CRISPR screens and GSE189764 [https://www.ncbi.nlm.nih.gov/geo/query/acc.cgi?acc=GSE189764] for the RNA-seq experiment.
The residual tumor data used in this study are available in the GEO database under accession codes:
GSE87455 [https://www.ncbi.nlm.nih.gov/geo/query/acc.cgi?acc=GSE87455] [PMID: 28940389],
GSE165252 [https://www.ncbi.nlm.nih.gov/geo/query/acc.cgi?acc=GSE165252] [PMID: 33504550],
GSE15781 [https://www.ncbi.nlm.nih.gov/geo/query/acc.cgi?acc=GSE15781] [PMID: 19969511],
GSE40442 [https://www.ncbi.nlm.nih.gov/geo/query/acc.cgi?acc=GSE40442] [PMID: 23297133].
The RPPA data in TCGA project used for survival analysis (TRGAted application) were from the TCPA portal [PMID: 30345029; PMID: 24037243] developed by MD Anderson Cancer Center. The remaining data are available within the article, supplementary information or source data file. All source data are included within the paper.

Human research participants

Policy information about studies involving human research participants and Sex and Gender in Research.

Reporting on sex and gender	NA
Population characteristics	NA
Recruitment	NA
Ethics oversight	NA

Note that full information on the approval of the study protocol must also be provided in the manuscript.

Field-specific reporting

Please select the one below that is the best fit for your research. If you are not sure, read the appropriate sections before making your selection.

☒ Life sciences ☐ Behavioural & social sciences ☐ Ecological, evolutionary & environmental sciences

For a reference copy of the document with all sections, see nature.com/documents/nr-reporting-summary-flat.pdf

Life sciences study design

All studies must disclose on these points even when the disclosure is negative.

Sample size	Sample sizes were determined empirically based on similar studies. For in vitro assays, n = 3-5. For in vivo animal studies, usually n = 3-5 for measurement of tumor size and n >= 12 for long-term survival analyses (PMID 35750880). The number of samples analyzed are indicated in the figure legends.
Data exclusions	No data were excluded from the analysis.
Replication	Key findings were repeated in independent experiments, as noted in the figure legends.
Randomization	For in vivo experiments, animals were randomly assigned to either control or treatment groups. For in vitro experiments, cells were randomly allocated into different treatment group.
Blinding	Researchers were blinded when collecting BLI data, with image acquisition by one staff member and data analysis by another one. For in vitro experiments, the researchers were blinded to cell genotypes or treatment conditions for imaging and analysis. For RNA-seq experiments, the analyst was blinded to treatment and genotype information.

Reporting for specific materials, systems and methods

We require information from authors about some types of materials, experimental systems and methods used in many studies. Here, indicate whether each material, system or method listed is relevant to your study. If you are not sure if a list item applies to your research, read the appropriate section before selecting a response.

Materials & experimental systems

n/a	Involved in the study
☐	☒ Antibodies
☐	☒ Eukaryotic cell lines
☒	☐ Palaeontology and archaeology
☐	☒ Animals and other organisms
☒	☐ Clinical data
☒	☐ Dual use research of concern

Methods

n/a	Involved in the study
☒	☐ ChIP-seq
☐	☒ Flow cytometry
☒	☐ MRI-based neuroimaging

Antibodies

Antibodies used

The antibodies: TSC1 (#6935), TSC2 (#4308), phospho-S6 (#4858), S6 (#2217), p21 (#2947), Lamin B1 (#12586), LC3 A/B (#12741), phospho-ATG14-S29 (#92340), ATG14 (#96752), phospho-CREB-S133 (#9198), CREB (#9197), phospho-CHK1-S345 (#2348), phospho-WEE1-S642 (#4910), WEE1(#13084), PLK1 (#4513), γH2A.X (#9718), phospho-S6K-S371 (#9208), S6K (#2708), phospho-4EBP1-S65 (#9451), 4EBP1 (#9644), phospho-AKT-S473 (#4060), AKT (#4691), p53 (#2527), phospho-CDC2-T15 (#9111), CDC2 (#9116), and phospho-ATR-S428 (#2853) from Cell signaling (all used at 1: 1000 dilution); β-tubulin (1: 4000, #10094-1-ap), β-actin (1: 4000, #20536-1-ap), and GAPDH (1: 10000, #10494-1-ap) from ProteinTech; ATR (1: 1000, #A300-137A-T) from Bethyl Lab, and CHK1 (1:1000, #sc-8408) and Cyclin B1(1: 1000, #sc-245) from Santa Cruz.

Validation

All the antibodies are commercially available and validated by the manufacturer:
TSC1: https://www.cellsignal.com/products/primary-antibodies/hamartin-tsc1-d43e2-rabbit-mab/6935
TSC2: https://www.cellsignal.com/products/primary-antibodies/tuberin-tsc2-d93f12-xp-rabbit-mab/4308
phospho-S6: https://www.cellsignal.com/products/primary-antibodies/phospho-s6-ribosomal-protein-ser235-236-d57-2-2e-xp-rabbit-mab/4858
S6: https://www.cellsignal.com/products/primary-antibodies/s6-ribosomal-protein-5g10-rabbit-mab/2217
p21: https://www.cellsignal.com/products/primary-antibodies/p21-waf1-cip1-12d1-rabbit-mab/2947
Lamin B1: https://www.cellsignal.com/products/primary-antibodies/lamin-b1-d4q4z-rabbit-mab/12586
LC3 A/B: https://www.cellsignal.com/products/primary-antibodies/lc3a-b-d3u4c-xp-rabbit-mab/12741
phospho-ATG14: https://www.cellsignal.com/products/primary-antibodies/phospho-atg14-ser29-d4b8m-rabbit-mab/92340
phospho-CREB: https://www.cellsignal.com/products/primary-antibodies/phospho-creb-ser133-87g3-rabbit-mab/9198
CREB: https://www.cellsignal.com/products/primary-antibodies/creb-48h2-rabbit-mab/9197
phospho-CHK1: https://www.cellsignal.com/products/primary-antibodies/phospho-chk1-ser345-133d3-rabbit-mab/2348
phospho-WEE1: https://www.cellsignal.com/products/primary-antibodies/phospho-wee1-ser642-d47g5-rabbit-mab/4910
WEE1: https://www.cellsignal.com/products/primary-antibodies/wee1-d10d2-rabbit-mab/13084
PLK1: https://www.cellsignal.com/products/primary-antibodies/plk1-208g4-rabbit-mab/4513
γH2A.X: https://www.cellsignal.com/products/primary-antibodies/phospho-histone-h2a-x-ser139-20e3-rabbit-mab/9718
phospho-S6K: https://www.cellsignal.com/products/primary-antibodies/phospho-p70-s6-kinase-ser371-antibody/9208
S6K: https://www.cellsignal.com/products/primary-antibodies/p70-s6-kinase-49d7-rabbit-mab/2708
phospho-4EBP1: https://www.cellsignal.com/products/primary-antibodies/phospho-4e-bp1-ser65-antibody/9451
4EBP1: https://www.cellsignal.com/products/primary-antibodies/4e-bp1-53h11-rabbit-mab/9644
phospho-AKT: https://www.cellsignal.com/products/primary-antibodies/phospho-akt-ser473-d9e-xp-rabbit-mab/4060
AKT: https://www.cellsignal.com/products/primary-antibodies/akt-pan-c67e7-rabbit-mab/4691
p53: https://www.cellsignal.com/products/primary-antibodies/p53-7f5-rabbit-mab/2527
phospho-CDC2: https://www.cellsignal.com/products/primary-antibodies/phospho-cdc2-tyr15-antibody/9111
CDC2: https://www.cellsignal.com/products/primary-antibodies/cdc2-poh1-mouse-mab/9116
phospho-ATR: https://www.cellsignal.com/products/primary-antibodies/phospho-atr-ser428-antibody/2853
β-tubulin: https://www.ptgcn.com/products/TUBB-Antibody-10094-1-AP.htm
GAPDH: https://www.ptglab.com/products/GAPDH-Antibody-10494-1-AP.htm
ATR: https://www.thermofisher.com/antibody/product/ATR-Antibody-Polyclonal/A300-137A
CHK1: https://www.scbt.com/p/chk1-antibody-g-4
Cyclin B1: https://www.scbt.com/p/cyclin-b1-antibody-gns1

Eukaryotic cell lines

Policy information about cell lines and Sex and Gender in Research

Cell line source(s)

The 4292 murine cell line was a generous gift from Dr. Marina Pasca di Magliano at University of Michigan. For human cell lines, MIA PaCa-2 (#85052806), PANC-1 (#87092802) and SCC47 (#SCC071) were from Sigma, MDA-MB-231 (#36) and SK-OV-3 (#43) were from MD Anderson, HeLa (#CCL-2), DU145 (#HTB-81), MeWo (#HTB-65), BxPC3 (#CRL-1687), Capan-2 (#HTB-80), LNCaP (#CRL-1740), C4-2B (#CRL-3315), 22Rv1 (#CRL2505), PC3 (#CRL-1435), T-47D (#HTB-133), MCF7 (#HTB-22), SK-BR-3 (#HTB-30), PLC/PRF/5 (#CRL-8024), SNU398 (#CRL-2233), Hep3B (#HB-8064), HepG2 (#HB-8065), A375 (CRL#1619), HCT116 (#CCL-247), SW480 (#CCL-228), H460 (#HTB-177), A549 (#CCL-185), H1299 (#CRL-5803), H441 (#HTB-174), U2OS (#HTB-96), AsPC-1 (#CRL1682) were from ATCC.

Authentication	Cell lines were authenticated by short tandem repeat profiling at the MD Anderson Cancer Center cell line core.
Mycoplasma contamination	All cells were tested as negative for mycoplasma contamination by PCR at the MD Anderson Cancer Center cell line core.
Commonly misidentified lines (See ICLAC register)	No misidentified cell lines were used in this study.

Animals and other research organisms

Policy information about studies involving animals; ARRIVE guidelines recommended for reporting animal research, and Sex and Gender in Research

Laboratory animals	NSG mice, 6-8 weeks old. All mice were maintained under standard conditions, at ambient temperature, 60% humidity, 12-hour light/dark cycles and received a standard diet and water ad libitum. Mice were euthanized by CO2 exposure followed by cervical dislocation when the tumor diameter reached 1.5cm, the maximal tumor size allowed by the IACUC committee.
Wild animals	No wild animals were used.
Reporting on sex	Equal number of male and female mice were used.
Field-collected samples	No field-collected samples were used.
Ethics oversight	All animal experiments were approved by IACUC at Houston Methodist Research Institute and performed in accordance with institutional and national guidelines.

Note that full information on the approval of the study protocol must also be provided in the manuscript.

Flow Cytometry

Plots

Confirm that:

- [x] The axis labels state the marker and fluorochrome used (e.g. CD4-FITC).
- [x] The axis scales are clearly visible. Include numbers along axes only for bottom left plot of group (a 'group' is an analysis of identical markers).
- [x] All plots are contour plots with outliers or pseudocolor plots.
- [x] A numerical value for number of cells or percentage (with statistics) is provided.

Methodology

Sample preparation	For cell cycle distribution, cells were resuspended in PBS, fixed with ice-cold ethanol, and treated with RNase and propidium iodide and analyzed on a BD FACS LSRII flow cytometer (BD Bioscience).<br>Quantification of SABG enzymatic activity followed a previously described protocol. Briefly, cells were treated with bafilomycin A1 (100nM) for one hour and stained with C12-FDG (33µM) for two hours at 37 degree under 5% CO2 before flowcytometric analysis.
Instrument	BD FACS LSRII flow cytometer (BD Bioscience).
Software	Data were collected using BD FACSDiva and analyzed with Flowjo (v10.8.1).
Cell population abundance	Not applicable. The cell cycle analysis and C-12 FDG assay did not use cell sorting.
Gating strategy	Population of interest was identified from a FSC-A and SSC-A plot to exclude the debris. For cell cycle analysis, the FL2-A and FL2-W plot were used to eliminate doublets. For C12-FDG assay, single cells were gated based on FSC-H and FSC-A.

- [x] Tick this box to confirm that a figure exemplifying the gating strategy is provided in the Supplementary Information.
